# Permeabilize, but Choose Wisely: Selective Antibiotic Potentiation Through Outer Membrane Disruption in *Pseudomonas aeruginosa*

**DOI:** 10.3390/ijms26209844

**Published:** 2025-10-10

**Authors:** Marine Novelli, Jean-Michel Brunel

**Affiliations:** Aix-Marseille Université, INSERM, SSA, MCT, 13385 Marseille, France; marine.novelli@etu.univ-amu.fr

**Keywords:** Gram-negative resistance, permeability barrier, antibiotic potentiation, *Pseudomonas aeruginosa*

## Abstract

Most clinically used antibiotics exert their effects by targeting essential intracellular components of bacterial cells. Therefore, enhancing their ability to traverse the bacterial envelope is crucial for restoring or improving therapeutic efficacy. We investigated the potential of outer membrane (OM)-disrupting agents—EDTA, NV716, colistin, and squalamine—to potentiate antibiotic activity against the multi-drug-resistant pathogen *Pseudomonas aeruginosa*. Our objective was to assess the therapeutic value of this strategy while also delineating its limitations by comparing responses across antibiotic classes with diverse chemical structures and pharmacological profiles. Beyond lipophilicity, we analyzed three additional physicochemical descriptors likely to influence OM permeability: molecular surface area, polarizability, and polar surface area. Our findings offer practical insights for the rational design of antibiotic–adjuvant combinations. While each descriptor provides valuable interpretive information, none alone reliably predicts OM-mediated potentiation. Instead, these factors should be viewed collectively within a multidimensional physicochemical profile, where optimal ranges of size, polarity, and lipophilicity act synergistically to enhance antibiotic uptake. By defining a shared multidimensional “responsive zone,” we propose a framework to guide the selection or design of antibiotics compatible with OM-disrupting strategies, potentially enabling the repurposing of antibiotics limited by poor OM permeability.

## 1. Introduction

Expanding the therapeutic arsenal against multidrug-resistant (MDR) bacteria represents a major challenge for the coming decades, especially in the light of alarming projections concerning antimicrobial resistance (AMR). According to a comprehensive study, an estimated 4.95 million deaths were associated with AMR in 2019, including 1.27 million directly attributable to drug-resistant infections [1]. This makes AMR as the third leading cause of death globally. Without effective intervention, projections indicate that AMR could claim up to 10 million lives annually by 2050 [2]. Despite notable advances in the treatment of Gram-positive bacterial infections, highlighted by the introduction of two new antibiotic classes in recent decades, namely cyclic lipopeptides (e.g., daptomycin) and oxazolidinones (e.g., linezolid), the development of novel agents targeting Gram-negative pathogens has stagnated significantly [3,4]. Predictive models suggest that to effectively address the escalating pace of resistance emergence, approximately twenty new classes will need to be developed within the next 50 years [5].

The stagnation in the development of antibiotics targeting Gram-negative bacteria is largely attributed to the intrinsic permeability barriers and active defense mechanisms of these organisms, which severely limit the intracellular accumulation of antimicrobial compounds [6]. The susceptibility to antibiotics is governed by a dynamic balance between two opposing molecular fluxes across the cell envelope: the passive influx through the outer membrane (OM) and the active efflux mediated by multidrug transporters [7]. The OM constitutes a formidable permeability barrier that restricts the entry of many antibiotics. Unlike the single-membrane structure of Gram-positive bacteria or the phospholipid bilayers of eukaryotic cells, the OM of Gram-negative bacteria is uniquely asymmetric. Its outer leaflet is densely packed with lipopolysaccharides (LPS), which confer rigidity and a strong negative surface charge, while the inner leaflet is composed of phospholipids, with non-specific porins and substrate-specific channels embedded in the membrane. This unique organization restricts the passive diffusion of lipophilic compounds, a relatively slow process, and limits the uptake of hydrophilic or charged molecules by size exclusion through narrow porins [8,9]. In parallel, Gram-negative bacteria have active efflux systems, such as those of the resistance–nodulation–division (RND) superfamily, which expel a broad range of antibiotics and toxic compounds before they can reach their targets [10]. Efflux pumps are thus key contributors to resistance mechanisms, and their activity has been shown to significantly reduce the intracellular concentration of many clinically relevant antibiotics [11,12]. The interplay between OM impermeability and active efflux establishes a highly effective, dual-layered defense system. These two mechanisms act synergistically, where even minor reductions in influx or increases in efflux can profoundly impact drug accumulation. Recent kinetic models and empirical data underscore how subtle changes in either flux can shift the balance and restore bacterial susceptibility to previously ineffective compounds [13,14]. To overcome these challenges, multiple strategies have been explored over the past few decades, including the use of efflux pump inhibitors [15], chemical modification of existing antibiotics to improve uptake or evade efflux [16,17,18], and agents capable of disrupting or permeabilizing the OM [19]. Collectively, these approaches aim to overcome the intrinsic defenses of Gram-negative bacteria and ultimately restore the efficacy of antibiotic treatments.

On the other hand, national surveillance data have revealed a concerning increase in the prevalence of MDR *Pseudomonas aeruginosa isolates* over recent years [20]. This opportunistic Gram-negative pathogen is particularly problematic due to its remarkable metabolic versatility and environmental adaptability, enabling its persistence in both community and healthcare environments. Vulnerable patient populations—including individuals with burn injuries, cystic fibrosis, acute leukemia, organ transplants, or those who use intravenous drugs—are especially susceptible to *P. aeruginosa* infections, which are often difficult to treat and associated with high rates of morbidity and mortality. These infections frequently lead to prolonged hospital stays and significantly increased healthcare costs [21,22]. Developing new antibiotics effective against *P. aeruginosa* presents a major challenge, largely due to its formidable cell envelope defenses. This bacterium possesses an intrinsically low-permeability outer membrane [23] and expresses multiple RND efflux pumps that work in concert to prevent antibiotic accumulation within the cell. These synergistic barriers severely limit the intracellular access of most compounds, including promising drug candidates. As a result, the hit rate for active molecules during compound library screenings is dramatically reduced, up to 1000-fold lower in *P. aeruginosa* than in Gram-positive bacteria [24]. This low success rate underscores the urgent need for innovative therapeutic approaches capable of circumventing or neutralizing these intrinsic resistance mechanisms. Strategies that enhance the permeability of the bacterial envelope and/or inhibit efflux activity are essential to improving antibiotic efficacy against *P. aeruginosa* and addressing the escalating public health threat it represents.

Most clinically used antibiotics exert their activity by targeting essential intracellular components of bacterial cells. Therefore, improving their ability to traverse the bacterial envelope is crucial to restoring or enhancing their efficacy [25]. Alterations in porin expression, modifications of channel selectivity, and structural changes in LPS further reduce OM permeability, complicating antibiotic entry. To overcome these barriers, various agents have been proposed to disrupt or destabilize the OM, thereby facilitating antibiotic penetration. Numerous studies have identified diverse classes of OM-disrupting compounds capable of sensitizing Gram-negative bacteria to antibiotics that would otherwise be ineffective. However, most research has focused predominantly on characterizing individual potentiator molecules, with limited attention given to the broader strengths and limitations of this strategy. A critical gap remains in our understanding of how the physicochemical properties of different antibiotic classes influence their ability to benefit from OM perturbation. Addressing this gap is essential to rationally guide the selection of antibiotic–potentiator combinations with optimal therapeutic potential.

In this study, we evaluate the capacity of OM-disrupting agents to enhance the activity of antibiotics against the MDR pathogen *P. aeruginosa*. We aim not only to assess the therapeutic promise of this strategy but also to delineate its limitations by comparing responses across diverse antibiotic classes with distinct chemical and pharmacological profiles.

## 2. Results

### 2.1. Potentiation of Antibiotics with OM Permeabilizers

**Figure 1:**
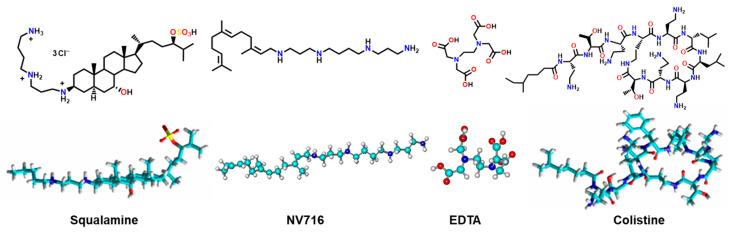
Structure of the selected potent antibiotic enhancers.

To explore whether the type of OM disruption influences antibiotic sensitivity in *P. aeruginosa*, we focused on four potentiators spanning the major categories of known OM disruptors: squalamine, the polyaminofarnesyl derivative NV716, the chelator ethylenediaminetetraacetic acid (EDTA), and the peptide colistin (Figure 1).

These structurally diverse molecules have previously been characterized by their distinct mechanisms of outer membrane perturbation. Squalamine is an aminosterol compound that integrates into the bacterial OM through electrostatic interactions with negatively charged lipids, leading to increased membrane permeability and loss of integrity [26]. NV716, a polyaminoisoprenyl derivative, binds to LPS and induces OM destabilization [27]. EDTA functions as a divalent cation chelator, extracting Ca^2+^ and Mg^2+^ ions that stabilize LPS-LPS interactions, thereby weakening membrane cohesion and increasing permeability [28]. Colistin, a last-resort polymyxin antibiotic, exerts its activity by displacing cationic bridges between LPS molecules via its polycationic peptide structure, followed by insertion of its hydrophobic tail into the membrane, ultimately leading to OM disruption and cell lysis [29,30]. To assess the impact of these enhancers, we screened a panel of 16 antibiotics (Figure S1) to evaluate their degree of potentiation under each condition, so we determined the minimum inhibitory concentrations (MICs) of these antibiotics with the four OM permeabilizers. This selection encompasses a broad spectrum of antibiotic classes—including β-lactams, fluoroquinolones, macrolides, rifamycins, tetracyclines, amphenicols, and glycopeptides—chosen to reflect diverse physicochemical properties, modes of action, and known outer membrane permeability profiles in *P. aeruginosa*, ranging from poorly permeating compounds due to their size, charge, or hydrophobicity (e.g., macrolides and rifampicin) to those with moderate to good intrinsic permeability (e.g., fluoroquinolones and β-lactams).

All results are summarized in Table 1. Globally, the OM permeabilizers enhanced the activity of several antibiotics, but the magnitude of potentiation varied substantially depending on both the antibiotic and the compound used. The concentrations of NV716 (10 µM), EDTA (1 mM), colistin (0.35 µM), and squalamine (5 µM) were selected based on published studies and preliminary assays, ensuring that they were below their individual MICs and had no intrinsic antibacterial activity on PAO1. A 4-fold or greater reduction in MIC was considered indicative of significant potentiation. Among the most striking effects, the tetracyclines (doxycycline, demeclocycline and minocycline) displayed strong MIC reductions in the presence of NV716 and EDTA. For example, the MIC of doxycycline decreased from 64 mg/L to 0.5 mg/L with NV716 (128-fold) and 1 mg/L with EDTA (64-fold). Similar patterns were observed for demeclocycline and minocycline, confirming the effectiveness of OM permeabilization in enhancing the activity of this class. The amphenicols (chloramphenicol and florfenicol), which typically display low activity in *P. aeruginosa*, also exhibited marked potentiation. Chloramphenicol MIC decreased from 64 mg/L to 4 mg/L with both NV716 and EDTA (16-fold), while florfenicol dropped from 256 mg/L to 4 mg/L with NV716 and 16 mg/L with EDTA. These data highlight the role of OM permeability in limiting the activity of amphenicols. Macrolides (azithromycin, dirithromycin, erythromycin) and rifampicin, which are generally inactive against *P. aeruginosa*, showed variable potentiation. Azithromycin MIC was reduced to 4-fold by NV716 (from 128 to 32 mg/L), while dirithromycin and erythromycin showed 2- to 4-fold changes depending on the compound. Rifampicin MICs dropped from 32 mg/L to 2 mg/L with NV716 and EDTA (16-fold), and even further to 1 mg/L with colistin (32-fold). Fluoroquinolones (ciprofloxacin and enrofloxacin) already exhibited low baseline MICs in contrast to nalidixic acid. Further potentiation was observed, especially with squalamine and NV716. Ciprofloxacin MIC was reduced 8-fold by squalamine (0.25 to 0.031 mg/L), and by nalidixic acid from 256 mg/L to 8 mg/L with NV716 (32-fold). Among β-lactams, ceftazidime and ticarcillin showed moderate but significant potentiation (4-fold) with EDTA only. In contrast, oxacillin intrinsically inactive against Gram-negatives, remained largely unaffected under all conditions. Interestingly, colistin and squalamine displayed narrower potentiation profiles compared to NV716 and EDTA. While colistin strongly enhanced rifampicin and slightly improved other antibiotics, squalamine primarily potentiated fluoroquinolones and chloramphenicol, but had minimal impact on macrolides or β-lactams.

Taken together, these results demonstrate that OM permeabilizers can significantly enhance the activity of several antibiotics against *P. aeruginosa*. However, the potentiation effect is highly dependent on the nature of both the antibiotic and the permeabilizer, and a 4-fold MIC reduction was not consistently achieved for all combinations. This suggests that OM permeabilization alone is not sufficient to guarantee improved antibiotic entry, and that physicochemical properties influence the ability of a compound to benefit from increased membrane permeability. In the following sections, we investigate how these parameters correlate with the degree of potentiation across different antibiotic classes.

### 2.2. Correlation Between Potentiation and Physicochemical Properties of Antibiotics

**Figure 2:**
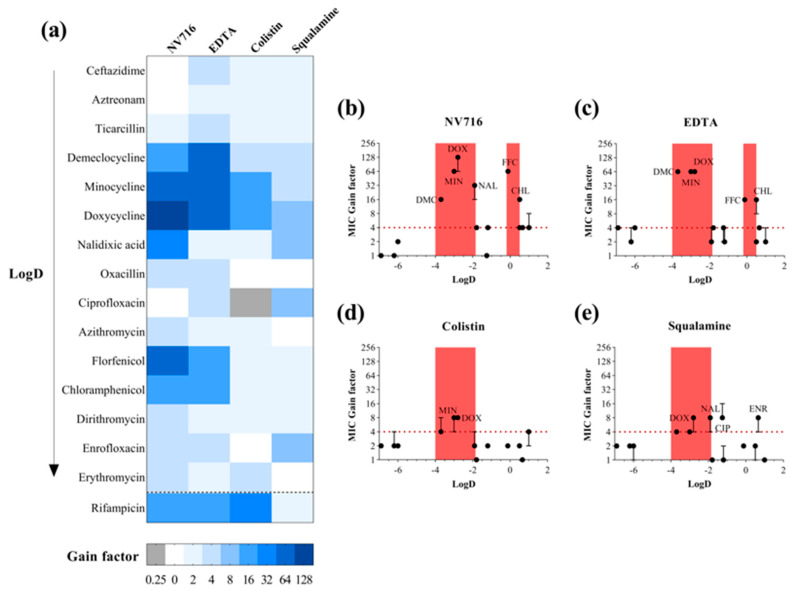
Enhancement of PAO1 antibiotic susceptibility by outer membrane permeabilizers. (**a**) Heatmap representation of PAO1 susceptibility gains for various antibiotics in combination with four outer membrane permeabilizers used as adjuvants: NV716 (10 µM), EDTA (1 mM), colistin (0.35 µM), and squalamine (5 µM). Adjuvant concentrations were selected to have no impact on PAO1 growth alone. The gain factor was calculated as: MIC of the antibiotic alone/MIC in combination with the adjuvant. Darker shades indicate a higher gain in susceptibility. A gain of ≥4-fold was considered significant. Antibiotics are ordered by increasing LogD values; (**b**–**e**) MIC gain factors (log scale) were plotted against the calculated LogD values of antibiotics in combination with the four outer membrane permeabilizers. The red dotted line marks the 4-fold gain threshold, considered a significant enhancement of antibiotic activity. Red areas highlight two LogD windows tested in this study: from −4 to −2 and from −0.2 to 0.5, where enhanced susceptibility was observed. Data points represent the median of three independent experiments; error bars indicate the range, where applicable.

Given the heterogeneous potentiation profiles observed across antibiotic classes, we next investigated whether specific physicochemical parameters could predict the ability of an antibiotic to benefit from OM permeabilization. For this analysis, we focused on four descriptors: calculated LogD at pH 7.4, molecular surface area (MSA), polarizability, and polar surface area (PSA) (Table S1, calculated by using Marvin sketch 20.6, default parameters). These parameters were selected to reflect key factors influencing OM penetration, including size, hydrophobicity, and polarity. We compared the extent of potentiation (expressed as MIC fold change in the presence of permeabilizers) with each of these molecular descriptors. The goal was to determine whether traits were associated with reduced permeation through the OM and, conversely, greater sensitivity to OM disruption. To better visualize the differential potentiation profiles across antibiotics and OM permeabilizers, we represented the MIC gain factors (i.e., fold-reduction in MIC) for each antibiotic–adjuvant combination in a heatmap (Figure 2a). The heatmap highlights the heterogeneous potentiation patterns observed, with NV716 and EDTA showing the broadest and most potent effects, particularly on tetracyclines (doxycycline, minocycline, demeclocycline), amphenicols (chloramphenicol, florfenicol), and certain hydrophilic agents such as nalidixic acid.

#### 2.2.1. A Lipophilicity Window for OM-Mediated Potentiation

To explore whether the lipophilicity of the antibiotics (as measured by LogD at pH 7.4) could explain their degree of potentiation, we plotted the MIC gain factors as a function of LogD values for each antibiotic–adjuvant combination (Figure 2b–e). A 4-fold reduction in MIC (red dashed line) was used as a threshold for significant potentiation. Across all four permeabilizers, a consistent pattern emerged: most significantly potentiated antibiotics clustered within a LogD range of −4 to −2. This red zone of potentiation includes tetracyclines (doxycycline, minocycline, demeclocycline) and nalidixic acid. In contrast, very hydrophilic compounds (LogD < −4, e.g., β-lactams) and highly hydrophobic antibiotics (LogD > 0, e.g., macrolides) generally exhibited limited or no potentiation, despite OM disruption. These observations suggest the existence of a “favorable lipophilicity window” in which molecules excluded by the intact OM become accessible upon membrane permeabilization. However, this correlation is not absolute, and exceptions were observed. Notably, NV716 and EDTA potentiated certain antibiotics with LogD values around 0, including chloramphenicol and florfenicol, suggesting that these adjuvants may extend the effective range of OM permeation. Squalamine also significantly potentiated ciprofloxacin and enrofloxacin (LogD −1.26 and 0.66, respectively), reinforcing the idea that individual adjuvant mechanisms can shift or broaden the effective physicochemical window. It is worth nothing that rifampicin was placed in a separate category, as it exhibits an atypical profile in terms of its LogD, which varies widely in the literature from 0.2 to 4.7 depending on the source [27,28]. Despite this variability, rifampicin shows strong synergy with NV716, EDTA, or colistin. Altogether, these results indicate that the LogD range from −4 to −2 represents a core zone of OM-permeabilizer responsiveness, conserved across diverse membrane-disrupting agents. Nevertheless, the presence of outliers highlights that lipophilicity alone is not sufficient to predict potentiation outcomes and that additional compound-specific factors modulate the extent of intracellular accumulation.

#### 2.2.2. Size, Polarity and Polarizability: Useful Filters but No Fixed Rules

**Figure 3:**
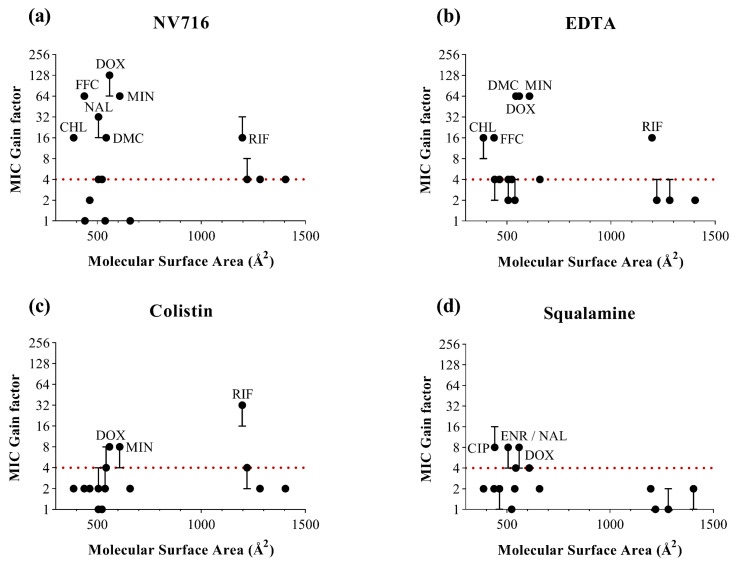
Relationship between the molecular surface area of antibiotics (in Å^2^) and MIC gain factor in PAO1 with the four OM permeabilizers. MIC gain factors (log scale) were plotted in the presence of (**a**) NV716 (10 µM); (**b**) EDTA (1 mM); (**c**) colistin (0.35 µM) and (**d**) squalamine (5 µM). The gain factor was calculated as the ratio between the MIC of the antibiotic alone and that in combination with the permeabilizer. The red dotted line indicates a 4-fold threshold for significant potentiation. Data points represent the median of three independent experiments; error bars indicate the range, where applicable.

To complement the analysis based on lipophilicity, we examined three additional physicochemical descriptors likely to influence OM permeability (Figure 3). For each parameter, MIC gain factors were plotted against the corresponding values for all antibiotics and OM permeabilizers. In contrast to LogD, no clear global correlation was observed across all permeabilizers and antibiotics. However, each descriptor offered complementary insights into structural features that may favor or limit OM-mediated uptake (Table 2).

For MSA (Figure 3), antibiotics with intermediate surface areas (400–700 Å^2^) tended to show consistent potentiation. These results support the idea that moderately sized molecules are well-positioned to benefit from OM permeabilization. However, this is not a sufficient condition, as several antibiotics within this size range dit not exhibit significant potentiation under most conditions. Conversely, rifampicin stands out as a notable exception: despite its large MSA (>1100 Å^2^), it was significantly potentiated by NV716, EDTA and colistin, with the last one yielding the strongest MIC reduction in the dataset. This observation indicates that a large MSA does not necessarily preclude intracellular accumulation, provided the OM is sufficiently compromised.

**Figure 4:**
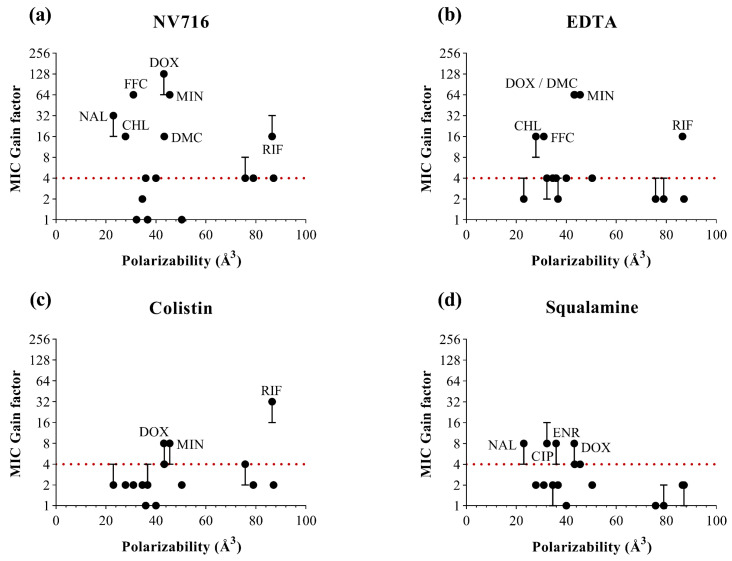
Relationship between the polarizability values of antibiotics (in Å^3^) and MIC gain factor in PAO1 with the four OM permeabilizers. MIC gain factors (log scale) were plotted in the presence of (**a**) NV716 (10 µM); (**b**) EDTA (1 mM); (**c**) colistin (0.35 µM) and (**d**) squalamine (5 µM). The gain factor was calculated as the ratio between the MIC of the antibiotic alone and that in combination with the permeabilizer. The red dotted line indicates a 4-fold threshold for significant potentiation. Data points represent the median of three independent experiments; error bars indicate the range, where applicable.

For polarizability (Figure 4), the most potent responses were generally associated with intermediate values (30–60 Å^3^), in line with several consistently potentiated antibiotics. This suggests that moderate polarizability may favor interactions with the perturbed membrane or facilitate passive diffusion through transient OM disruptions. However, outliers were again observed, and some highly polarized molecules (e.g., macrolides) remained poorly potentiated, highlighting the influence of additional structural constraints.

**Figure 5:**
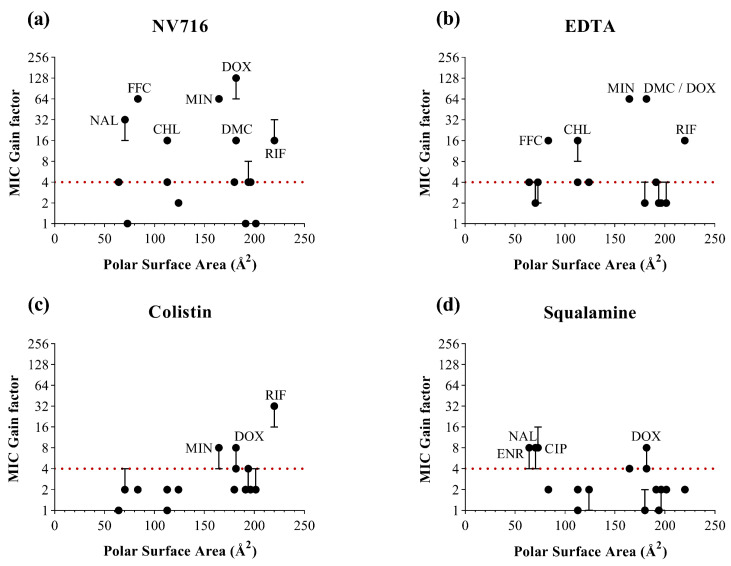
Relationship between the polar surface area of antibiotics (in Å^2^) and MIC gain factor in PAO1 with the four OM permeabilizers. MIC gain factors (log scale) were plotted in the presence of (**a**) NV716 (10 µM); (**b**) EDTA (1 mM); (**c**) colistin (0.35 µM) and (**d**) squalamine (5 µM). The gain factor was calculated as the ratio between the MIC of the antibiotic alone and that in combination with the permeabilizer. The red dotted line indicates a 4-fold threshold for significant potentiation. Data points represent the median of three independent experiments; error bars indicate the range, where applicable.

In contrast, for PSA (Figure 5), no consistent potentiation window could be identified across the four permeabilizers. Antibiotics with both low and high PSA values showed heterogeneous responses. These findings suggest that PSA alone does not predict the extent of potentiation following OM permeabilization. An exception was observed with colistin, which appeared to preferentially potentiate antibiotics with higher PSA values, such as rifampicin. This may reflect a specific mechanism of OM disruption by colistin, favoring the uptake of more polar or hydrophilic structures once the barrier is compromised.

Altogether, these observations indicate that while individual descriptors such as MSA, polarizability, or PSA can provide useful interpretive elements, none alone defines a generalized rule for OM-mediated potentiation. These parameters must be considered as part of a multidimensional profile, where favorable ranges in size, polarity, and lipophilicity combine, rather than individually predict, the likelihood of potentiation.

## 3. Discussion

The impermeability of the Gram-negative OM represents a critical obstacle in the development and optimization of antibiotic therapies. In this study, we assessed the potentiating effect of four OM-disrupting agents (NV716, EDTA, colistin and squalamine) on a chemically diverse panel of antibiotics in *P. aeruginosa*. Our aim was not to identify effective combinations but to determine whether specific physicochemical properties could explain or predict susceptibility to OM-mediated potentiation.

Our analysis revealed that most antibiotics significantly potentiated by OM permeabilization clustered within a LogD range of −4 to −2, regardless of the permeabilizer used. This suggests the existence of a favorable lipophilicity window in which antibiotics are normally excluded by the intact OM but become permeable once the barrier is compromised. However, our study also revealed multiple exceptions, antibiotics within this LogD window that were not potentiated, and others with higher or lower LogD values that were. Thus, lipophilicity alone is not sufficient to predict permeabilization-induced potentiation.

To gain additional insights, we analyzed three further parameters. Antibiotics with intermediate MSA values (400–700 Å^2^) tended to be potentiated. However, this was not a universal rule: some moderately sized antibiotics were not potentiated, and rifampicin, despite its very high MSA (>1100 Å^2^), showed robust potentiation. Similarly, antibiotics with moderate polarizability values (30–60 Å^3^) were frequently potentiated, suggesting that polarizability may influence interactions with a destabilized OM. Again, outliers exist, indicating that polarizability, while informative, is not deterministic. For PSA, no consistent potentiation pattern emerged across permeabilizers. Interestingly, colistin seemed to favor antibiotics with higher PSA values, such as rifampicin. This may reflect colistin’s distinct mechanism of OM disruption, which involves displacement of divalent cations and deep penetration into the LPS layer, thereby facilitating entry of more polar compounds [30]. Overall, these trends reinforce that no single descriptor governs OM permeability. Rather, potentiation appears to arise from a combinatorial effect of several balanced molecular properties.

Our data support a multidimensional compromise model for OM-mediated potentiation. Antibiotics that fall within moderate ranges of lipophilicity, molecular surface area, and polarizability are the most likely to be potentiated by OM disruption. This idea aligns with broader efforts to map the chemical space compatible with antibiotic activity in Gram-negative bacteria [31,32].

Among the most effective permeabilizers, such as NV716 and EDTA, differences in their mechanisms of action are evident, despite both ultimately leading to OM disruption. NV716, in particular, has been shown to interact with LPS at sub-MIC, increasing the permeability of the OM in *P. aeruginosa* [27]. Additionally, it appears to inhibit efflux transporter activity through an as-yet unidentified mechanism, thereby enhancing antibiotic activity. On the other hand, the antimicrobial activity of EDTA has been well demonstrated against Gram-negative bacteria, whose outer membrane integrity is maintained by hydrophobic interactions among LPS molecules and by LPS–protein interactions [33,34]. Divalent cations such as Mg^2+^ and Ca^2+^ play a crucial role in stabilizing the negative charges of the LPS oligosaccharide chains. EDTA acts by chelating these cations, effectively disrupting the outer membrane [35,36]. This chelation leads to the release of up to 50% of LPS molecules, exposing the phospholipid-rich inner membrane and thereby enhancing the activity of other antimicrobials. Finally, although colistin and squalamine are both classified as cationic amphipathic compounds, previously reported to belong to the same family, they exhibit significant differences in their mechanisms of interaction with biological membranes. These differences may be tentatively attributed to variations in their polar-to-apolar balance, which influences their ability to penetrate lipid bilayers and induce electrically active membrane lesions. Notably, colistin demonstrates faster kinetics of membrane permeabilization and greater selectivity for bacterial cells compared to squalamine. This higher bacterial selectivity may stem from colistin’s superior capacity to insert into biological membranes and induce membrane destabilization and/or rupture.

## 4. Material and Methods

**Drugs.** All compounds were purchased from Sigma-Aldrich (Saint Quentin Fallavier, France) in pure powder form. Stock solutions of aztreonam, colistin, ceftazidime, EDTA, demeclocycline, doxycycline, minocycline, oxacillin, and ticarcillin were prepared in water. Nalidixic acid, ciprofloxacin, and enrofloxacin were dissolved in water with the addition of 30% sodium hydroxide. Chloramphenicol and florfenicol were solubilized in 70% ethanol. Azithromycin, dirithromycin, and erythromycin were dissolved in absolute ethanol. Rifampicin was dissolved in dimethyl sulfoxide. NV716 and squalamine were synthesized according to previously reported procedures in more than 95% pure form and dissolved in water as their hydrochloride salts [37,38].

**Software.** Software Marvin sketch version 20.6 was used for LogD calculation at pH 7.4 involving default settings parameters.

**Susceptibility testing.** Minimum inhibitory concentrations were determined for *P. aeruginosa* PAO1 using the standardized broth microdilution method, following EUCAST guidelines [39]. Experiments were carried out in cation-adjusted Mueller–Hinton II broth using sterile 96-well microplates, with twofold serial dilutions of the tested antibiotics. The preculture of PAO1 was grown to the exponential phase, and the bacterial suspension was adjusted to 1 × 10^6^ CFU/mL. Wells were inoculated to yield a final bacterial concentration of 5 × 10^5^ CFU/mL in a total volume of 200 μL. Outer membrane permeabilizers were added at fixed concentrations that did not affect bacterial growth. Plates were incubated at 37 °C for 18 h, and MICs were defined as the lowest concentration preventing visible bacterial growth. To confirm visual readings, iodonitrotetrazolium chloride (0.2 mg/mL) was added and plates were incubated at room temperature for 30 min; absence of a pink color indicated growth inhibition. MIC values are reported as the median of at least three independent experiments.

## 5. Conclusions

Our study offers practical insights for designing rational antibiotic–adjuvant combinations. By identifying a shared multidimensional “responsive zone,” we propose a framework to guide the selection or design of antibiotics compatible with OM-disrupting strategies. This could accelerate the repurposing of antibiotics that fail to cross the OM under normal conditions but remain active once the barrier is breached. However, our analysis does not account for efflux, target binding, or intracellular degradation, all of which influence antimicrobial efficacy. Integrating real-time accumulation assays, efflux profiling, and computational modeling will be crucial to developing a more predictive, quantitative understanding of antibiotic access in Gram-negative pathogens. Ongoing studies are currently being conducted and will be reported in due course.

## Figures and Tables

**Table 1:** Minimum inhibitory concentrations (mg/L) of PAO1 to selected antibiotics with or without outer membrane permeabilizers.

Antibiotic	MIC (mg/L)				
	Ø	+SQ (5 µM)	+NV716 (10 µM)	+EDTA (1 mM)	+CST (0.35 µM)
AZM	128	128	32	64	64
ATM	8	4	8	4	4
CAZ	2	1	2	0.5	1
CHL	64	32	4	4	32
CIP	0.25	0.031	0.25	0.0625	0.5
DMC	32	8	2	0.5	8
DTM	512	256	128	256	256
DOX	64	8	0.5	1	8
ENR	1	0.125	0.25	0.25	1
ERY	256	256	64	128	64
FFC	256	128	4	16	128
MIN	32	8	0.5	0.5	4
NAL	256	16	8	64	64
OXA	2048	2048	512	512	2048
RIF	32	16	2	2	1
TIC	32	16	16	8	16

AZM, azithromycin; ATM, aztreonam; CAZ, ceftazidime; CHL, chloramphenicol; CIP, ciprofloxacin; CST, colistin; DMC, demeclocycline; DTM, dirithromycin; DOX, doxycycline; ENR, enrofloxacin; ERY, erythromycin; FFC, florfenicol; MIN, minocycline; NAL, nalidixic acid; OXA, oxacillin; RIF, rifampicin; SQ, squalamine; TIC, ticarcillin, +: means in the presence of compounds; Ø means no presence of another compound added.

**Table 2:** Physicochemical properties of the antibiotics used in this study.

Antibiotic	Molecular Weight (g/mol)	Molecular Surface Area (Å^2^)	Calculated LogD at pH 7.4	Polarizability (Å^3^)	Polar Surface Area (Å^2^)
AZM	749	1282.33	−1.2	79.01	180.08
ATM	435.4	537.79	−6.2	36.66	201.58
CAZ	546.6	657.8	−6.9	50.38	191.22
CHL	323.13	386.28	0.5	27.82	112.7
CIP	331.34	440.5	−1.26	32.27	72.88
DMC	464.9	543.25	−3.7	43.4	181.62
DTM	835.1	1404.77	0.5	87.14	196.33
DOX	444.4	558.6	−2.8	43.22	181.62
ENR	359.4	505.82	0.66	35.93	64.09
ERY	733.9	1220.13	1.0	75.76	193.91
FFC	358.2	437.95	−0.12	31.01	83.47
MIN	457.5	607.96	−3.0	45.54	164.63
NAL	232.23	505.82	−1.9	23.02	70.5
OXA	401.4	523.19	−1.8	40	112.74
RIF	822.9	1197.16	3.5	86.48	220.15
TIC	384.4	463.93	−6.0	34.58	124.01

AZM, azithromycin; ATM, aztreonam; CAZ, ceftazidime; CHL, chloramphenicol; CIP, ciprofloxacin; DMC, demeclocycline; DTM, dirithromycin; DOX, doxycycline; ENR, enrofloxacin; ERY, erythromycin; FFC, florfenicol; MIN, minocycline; NAL, nalidixic acid; OXA, oxacillin; RIF, rifampicin; TIC, ticarcillin.

## Data Availability

The original contributions presented in this study are included in the article/Supplementary Material. Further inquiries can be directed to the corresponding author.

## Supplementary Materials

The following supporting information can be downloaded at: https://www.mdpi.com/article/10.3390/ijms26209844/s1.

## Abbreviations

The following abbreviations are used in this manuscript:

AMR	Antimicrobial Resistance
EDTA	Ethylene Diamine Tetra-acetic Acid
LPS	Lipopolysaccharides
MDR	Multi-Drug-Resistant
MIC	Minimum Inhibitory Concentration
MSA	Molecular Surface Area
OM	Outer Membrane
PSA	Polar Surface Area
RND	Resistance–Nodulation–Division
