# Supplementary figures and images for: Permeabilize, but Choose Wisely: Selective Antibiotic Potentiation Through Outer Membrane Disruption in Pseudomonas aeruginosa
Source: Int J Mol Sci. 2025 Oct 10;26(20):9844. doi: 10.3390/ijms26209844 (PMC12563807; doi:10.3390/ijms26209844)

**Figure S1.** Structure of the antibiotics used in this study

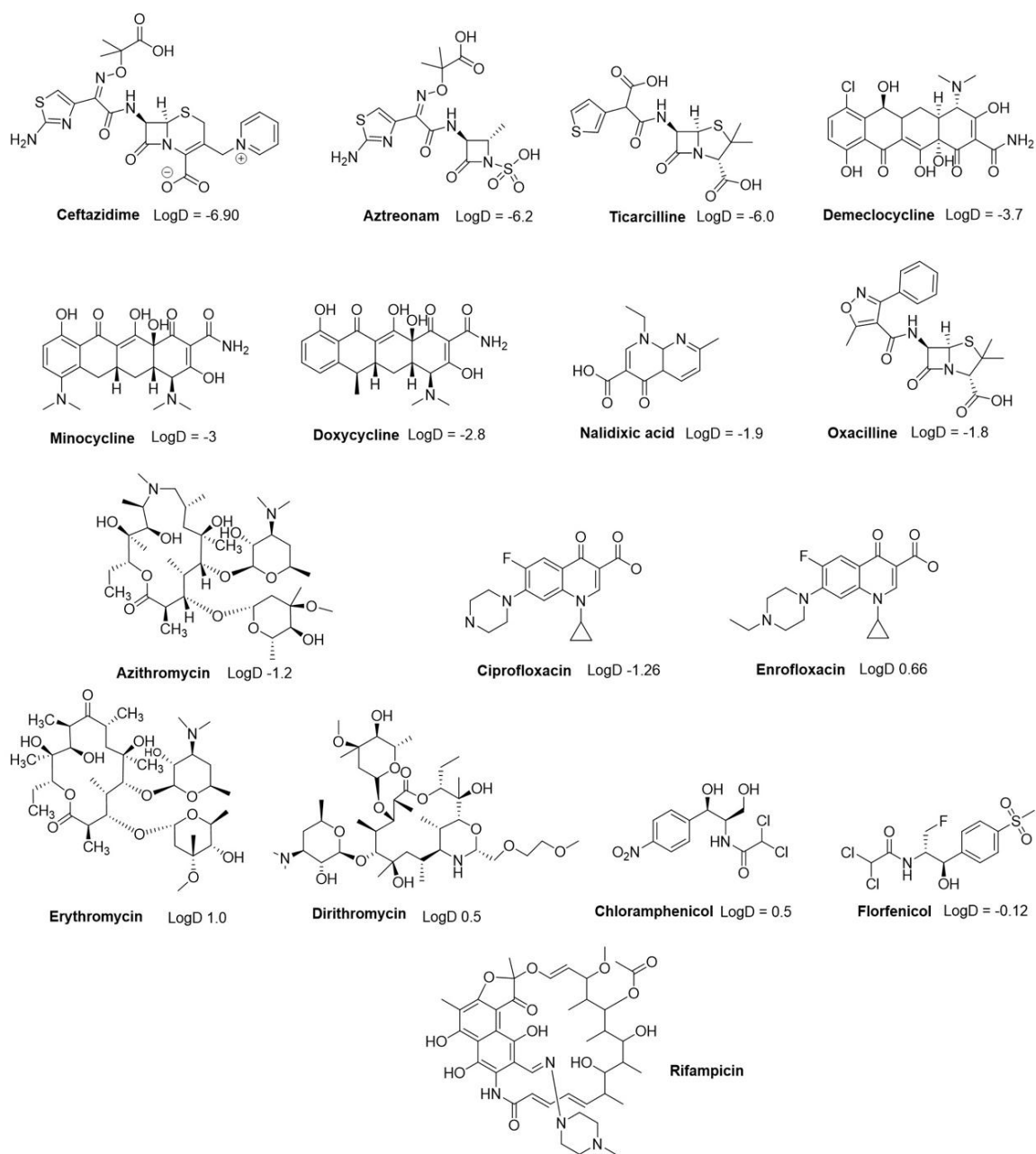

Supplement: Supplementary file 1 [file ijms-26-09844-s001.zip › ijms-3835411-supplementary.pdf]
